# Dietary Nitrate and Diet Quality: An Examination of Changing Dietary Intakes within a Representative Sample of Australian Women

**DOI:** 10.3390/nu10081005

**Published:** 2018-08-01

**Authors:** Jacklyn K. Jackson, Amanda J. Patterson, Lesley K. MacDonald-Wicks, Catherine P. Bondonno, Lauren C. Blekkenhorst, Natalie C. Ward, Jonathan M. Hodgson, Julie E. Byles, Mark A. McEvoy

**Affiliations:** 1 Faculty of Health and Medicine, School of Health Sciences, Department of Nutrition and Dietetics, University of Newcastle, Callaghan, NSW 2308, Australia; Jacklyn.Jackson@uon.edu.au (J.K.J.); Amanda.Patterson@newcastle.edu.au (A.J.P.); Lesley.Wicks@newcastle.edu.au (L.K.M.-W.); 2 Priority Research Centre for Physical Activity and Nutrition, University of Newcastle, Callaghan, NSW 2308, Australia; 3 Medical School, Royal Perth Hospital Unit, University of Western Australia, Perth, WA 6009, Australia; c.bondonno@ecu.edu.au (C.P.B.); l.blekkenhorst@ecu.edu.au (L.C.B.); Natalie.Ward@curtin.edu.au (N.C.W.); jonathan.hodgson@ecu.edu.au (J.M.H.); 4 School of Medical and Health Sciences, Edith Cowan University, Joondalup, WA 6207, Australia; 5 School of Public Health and Curtin Health Innovation Research Institute, Curtin University, Perth, WA 6102, Australia; 6 Research Centre for Gender, Health and Ageing, School of Medicine and Public Health, Faculty of Health and Medicine, Hunter Medical Research Institute, University of Newcastle, New Lambton, NSW 2305, Australia; Julie.byles@newcastle.edu.au; 7 Faculty of Health and Medicine, School of Medicine and Public Health, Hunter Medical Research Institute, University of Newcastle, New Lambton, NSW 2305, Australia

**Keywords:** dietary nitrate, diet quality, women

## Abstract

Dietary nitrate is increasingly linked to a variety of beneficial health outcomes. Our purpose was to estimate dietary nitrate consumption and identify key dietary changes which have occurred over time within a representative sample of Australian women. Women from the 1946–1951 cohort of the Australian Longitudinal Study on Women’s Health with complete food frequency questionnaire data for both 2001 and 2013 were included for analysis. Dietary nitrate intakes were calculated using key published nitrate databases. Diet quality scores including the Australian Recommended Food Score, the Mediterranean Diet Score and the Nutrient Rich Foods Index were calculated along with food group serves as per the Australian Dietary Guidelines. Wilcoxon matched pairs tests were used to test for change in dietary intakes and Spearman’s correlations were used to examine associations. In our sample of 8161 Australian women, dietary nitrate intakes were on average 65–70 mg/day, and we detected a significant increase in dietary nitrate consumption over time (+6.57 mg/day). Vegetables were the primary source of dietary nitrate (81–83%), in particular lettuce (26%), spinach (14–20%), beetroot (10–11%), and celery (7–8%) contributed primarily to vegetable nitrate intakes. Further, increased dietary nitrate intakes were associated with improved diet quality scores (*r* = 0.3, *p* < 0.0001). Although there is emerging evidence indicating that higher habitual dietary nitrate intakes are associated with reduced morbidity and mortality, future work in this area should consider how dietary nitrate within the context of overall diet quality can facilitate health to ensure consistent public health messages are conveyed.

## 1. Introduction

There is a growing body of evidence suggesting that dietary nitrate may have a protective role in health, in particular vascular health [1]. This is linked to the enterosalivary nitrate-nitrite-nitric oxide pathway, in which dietary nitrate can be metabolized in the human body to produce nitric oxide (NO) [2,3]. NO is an important chemical messenger, responsible for a variety of physiological functions including maintaining vascular health, gastrointestinal function, and regulation of immune and inflammatory cells [4,5].

Coronary heart disease (CHD), stroke, and heart failure are three of the leading causes of death among Australian women [6]. Cardiovascular Diseases (CVDs) are responsible for more than one-quarter of premature deaths in this group; more than 90% of Australian women have at least one risk factor for CVD; and risk substantially increases with age [7]. Despite this, much of the burden of CVD is preventable, and improved diet quality is a major global target to tackle this disease.

The cardio protective effects of vegetable consumption within the context of overall diet quality and specific dietary patterns including the Mediterranean diet, the traditional Japanese Diet, the Dietary Approaches to Stop Hypertension (DASH) diet, and vegetarian/plant-based diets are increasingly clear [8,9,10,11,12]. It is of interest to note that vegetables are a major source of dietary nitrate. In particular, rich sources include green leafy vegetables, beetroot, and celery, as nitrate tends to accumulate in the leaves, stems, and roots of plants [13].

Traditionally, dietary nitrate has been considered to be biologically unnecessary or a potentially harmful component of the food and water supply [14]. As a result it is strictly regulated, and is not included as part of National food composition tables in Australia [15,16]. This is despite the fact that the Food and Agriculture Organization has indicated a lack of direct evidence demonstrating that dietary nitrate and nitrite are carcinogenic to humans, and the recent discovery of the health promoting mechanisms associated with dietary nitrate [17,18]. Further, epidemiological studies have shown a high nitrate ingestion primarily from vegetables reduced the risk of gastric cancer, while other studies of nitrate and nitrite ingestion from processed meats found an increased risk [19]. These findings can be explained as carcinogenic *N*-nitroso compound formation is accelerated by the presence of factors including heme iron (common to meat) and inhibited by vitamin C and other antioxidants (common to vegetables) [19], suggesting that dietary nitrate intakes within the context of overall diet quality could be an important consideration when exploring dietary nitrate intakes and health.

A systematic review and meta-analysis investigating the short term effects of inorganic/dietary nitrate and CVD risk factors, was recently published [20]. Results of the meta-analysis of randomized controlled trials found that inorganic/dietary nitrate intakes can significantly reduce resting blood pressure (systolic blood pressure: −4.80 mmHg, *p* < 0.0001; diastolic blood pressure: −1.74 mmHg, *p* = 0.001), improve endothelial function (flow mediated dilatation: 0.59%, *p* < 0.0001), reduce arterial stiffness (pulse wave velocity: −0.23 m/s, *p* < 0.0001, augmentation index: −2.1%, *p* = 0.05) and reduce platelet aggregation by 18.9% (*p* < 0.0001) [20].

A large number of experimental trials have been performed within this research space. They generally indicate an acute/short-term beneficial effect of dietary/inorganic nitrate in doses greater than 130 mg/day [20]. However, the evidence base around long-term habitual intakes of dietary nitrate and CVD outcomes is still developing, and lower intakes might also be beneficial. 

In a world first, Blekkenhorst et al., was able to show that higher nitrate intakes (≥53 mg/day) were inversely associated with lower atherosclerotic vascular disease mortality in a cohort of older women (*n* = 1226) [21]. In the same cohort, inverse associations were also observed with vegetable-derived nitrate and common carotid artery intima-media thickness (as a marker of subclinical atherosclerosis), as well as ischemic cerebrovascular disease events [22].

Dietary nitrate is increasingly thought to have a role in facilitating the health promoting effects of vegetables and cardioprotective dietary patterns such as the Mediterranean diet [23]. In addition, it has been recently hypothesized that dietary nitrate intakes produce greater bioactive effects when consumed within the context of a healthy diet, rich in antioxidants and polyphenols which are thought to enhance the potency and bioactivity of dietary nitrate [24]. However, currently it is unknown how much dietary nitrate the Australian population is consuming. In addition, due to a general lack of reliable dietary data available in Australia, little is known about how Australians eating behaviors have changed over time, as they age. These are important questions to explore if we want to effectively improve overall diet quality through targeted large scale nutrition interventions. 

Therefore, the purpose of the current investigation was to estimate dietary nitrate consumptions within the context of overall diet quality, and identify key dietary changes which have occurred over time within a representative sample of Australian women.

## 2. Materials and Methods 

### 2.1. The Australian Longitudinal Study on Women’s Health (ALSWH)

The ALSWH (also known as Women’s Health Australia) was established in 1996 to investigate the health and wellbeing of Australian women over 20+ years of follow-up. At baseline in 1996, women in three age groups (1973–1978 cohort, 1946–1951 cohort, and 1921–1926 cohort) were randomly selected from the Medicare (Australia’s government funded universal healthcare cover) database [25]. Women living in rural and remote areas were intentionally oversampled to allow sufficient power to analyze data by area of residence [26]. However, the baseline sample of 40,000+ women recruited to the ALSWH were found to be a nationally representative sample [27].

Data used in the current study were derived from the 1946–1951 cohort of the ALSWH. Survey 1 was conducted in 1996, and women of this cohort were then surveyed every 2–3 years [27]. In total, data from two food frequency questionnaires (FFQ) conducted in 2001 and 2013 were included in the current study.

The Human Research Ethics Committee of the University of Newcastle and the University of Queensland approved the study methods. Further details on the sample and methods used by the ALSWH have been reported elsewhere and are available at www.alswh.org.au.

### 2.2. Assessment of Dietary Intake

Dietary intake was assessed using a FFQ known as the Dietary Questionnaire for Epidemiological Studies (DQES), version 2 [28]. The DQES was included as part of Survey 3 and Survey 7 only, collected in 2001 and 2013, respectively, from all participants. The DQES asked respondents to report their usual consumption of 74 foods and beverages and six alcoholic beverages over the preceding 12 months, using a 10-point frequency option from “Never” up to “3 or More times per day”. Portion size photographs were used to adjust the serve size for vegetables, meat and casseroles. Additional questions were asked about total number of daily serves of fruit, vegetables, bread, dairy products, eggs, fat spreads, and sugars, as well asking the type of bread, dairy products, and fat spread used. Both the development of the DQES and its validation in a sample of Australian women have been previously reported [29].

Only women with complete FFQ data at both time points (2001 (*n* = 10,629, 95% the cohort in 2001) and 2013 (*n* = 9115, 99% of the cohort in 2013)) were included for analysis (*n* = 8161). A sensitivity analysis was conducted in which women reporting extreme energy intakes (<2500 kJ/day and >20,000 kJ/day) were excluded from the analysis.

To ascertain if dietary nitrate intakes were correlated with overall dietary intakes, we calculated food group serves as specified by the Australian Dietary Guidelines (ADG) [30]. In addition, various measures of diet quality were calculated including the Nutrient Rich Food (NRF) index, the Australian Recommended Food Score (ARFS) [31], and the Mediterranean Diet Score (MDS) [32].

The ADG defines a standard serve of vegetables as approximately 75 g. Therefore, to calculate daily vegetable serves, the total grams of daily vegetable intake was divided by 75. Further, the ADG defines a standard serve of fruit as about 150 g, thus fruit serves were calculated by dividing the total grams of daily fruit intake by 150. The weight of a standard serve for dairy foods, grain foods, meat and/or meat alternatives, and discretionary choice foods were more varied based on the specific food item within these food groups, and therefore serves were calculated on a food item basis. For example, a serve of dairy from milk was calculated by dividing total daily grams of milk intake by 250, as a standard serve of milk is approximately 250 g. However, to calculate daily serves of dairy contributed from cheese, the ADG defines a standard serve of cheese as 40 g [30]. Once serves per food item were derived, foods within the same food group were added to calculate the total serves per day for that of food group. Food groups were calculated so that direct comparisons could be made between reported intakes and benchmarked against the National recommended dietary intakes.

Responses from the DQES were converted into average daily intakes, and individual food items were calculated in grams per day. Nutrient values were derived based on Australian nutrient composition data from NUTTAB (NUTrient TABles for use in Australia) [15].

The NRF index assesses diet quality by calculating dietary nutrient density scores. This index was calculated using Nutrient Reference Values (NRVs) for Australian and New Zealand based on adult women older than 50 years and younger than 70 years. The methods for calculating the NRF index have been reported in detail elsewhere, and criteria detailed in Supplementary Table S1 [21]. Briefly, the NRF index was calculated based on the percentage of adherence to the reference values for positive nutrients with associated health benefits (protein; fiber; vitamins A, C, and E; calcium; iron; potassium; and magnesium) and negative nutrients which should be limited (sodium, saturated fat, and added sugar). The NRF index was also standardized for energy intakes per 1000 kJ/day.

The ARFS assesses diet quality by calculating consistency with national recommendations to the Dietary Guidelines for Australian adults and the core foods outlined in the Australian Guide to Healthy Eating, where a higher ARFS score represents greater adherence to the ADG and better diet quality [31]. For example, FFQ items including fruits and vegetables consumed less than once a week scored zero and those consumed once a week or more, scored one. While meat FFQ items scored zero if consumed less than once a month or 5 or more times per week, and scored 1 if consumed 1–4 times per week. Additional points were also awarded for type and quantity of core food intake consistent with national dietary intake recommendations. A maximum of two points were added for alcohol consumption: one point for moderate frequency (up to 4 days/week) and the second point for moderate quantity (1 or 2 standard drinks, when alcohol was consumed). The maximum ARFS possible is 74, which was the total of the food group based sub-scores, including vegetable score (maximum score of 22), fruit score (maximum score of 14), grain score (maximum of 14), dairy score (maximum of 7), nuts/beans/soy/egg score (maximum of 7), meat score (maximum of 5), fish score (maximum of 2), fat score (maximum of 1), and alcohol score (maximum of 2 points). More detailed explanation for calculating the ARFS has been described elsewhere [31], and criteria outlined in Supplementary Table S2.

It has been suggested that dietary nitrate could in part be responsible for the cardiovascular benefits attributed from a Mediterranean diet, which is characterized by a high consumption of fruits, vegetable, legumes, cereals, fish, and olive oil, and a low consumption of milk and meat, and a moderate consumption of alcohol [32]. The Mediterranean diet score (MDS) was calculated as per Stefler et al. with one modification [32]. Olive oil consumption is not collected as part of the DQES. Therefore the MDS scoring was modified to match the information within the DQES, and the MDS fat component score was awarded if women reported consuming mono-unsaturated fat spreads. The maximum MDS possible was 17, criteria for calculating the MDS has been detailed in Supplementary Table S3.

### 2.3. Calculating Dietary Nitrate Intakes 

Nitrate data is not included within National food composition tables, therefore nitrate intakes were estimated based on published nitrate databases [13,33,34,35].

Vegetable nitrate data were derived from a recently published database, which includes worldwide vegetable nitrate data from 255 publications for up to 180 vegetables and 22 herbs and spices [13]. The previous application of this vegetable nitrate database found a positive correlation between urinary nitrate and 24-hour diet recalls (*r* = 0.4, *p* = 0.013) and application to the DQES was found to be moderately positively correlated with 24-hour diet recalls (*r* = 0.50, *p* < 0.001) [13]. Vegetable nitrate data from Blekkenhorst et al. [13], was applied to 24 of the DQES items.

Non-vegetable nitrate data were derived from three other key publications, in that nitrate values for 67 DQES items were obtained from Inoue-Choi et al. [33], five were obtained from the Food Standards Australia New Zealand survey of nitrates and nitrites in food and beverages in Australia [34], and nitrate values for three DQES items were obtained from Griesenbeck et al. [35]. When food nitrate data were available for FFQ items, the nitrate content of foods were calculated by multiplying the food item in grams by the nitrate content (mg) per gram.

### 2.4. Analysis

All calculations and analyses were conducted using Stata 14.2 (StataCorp, College Station, TX, USA). 

FFQ data were available at only two time points (2001 and 2013), therefore data for total dietary nitrate intake and nitrate consumption by food group (vegetables, fruit, meat, dairy, grains, alcohol, and discretionary choices) at each time point have been presented. Due to the skewed nature of the FFQ data, Wilcoxon matched pairs tests were performed to identify if there was a statistically significant change in dietary intakes of specific nutrients, food consumption, and/or diet quality over time.

Spearman’s rank correlation coefficients were conducted using “spearman” command, to assess if total nitrate consumption was associated with other dietary behaviors including diet quality (NRF index, ARFS, and MDS), total vegetable intake (g/day), and total green leafy vegetable intake (g/day).

## 3. Results

In a representative sample of 8161 Australian women, subtle, but statistically significant differences in dietary intakes were detected when comparing self-reported FFQ intakes in 2001 (women aged 50–55 years) to FFQ data reported in 2013 (women aged 62–67 years). 

Intakes of food groups as per the Australian Dietary Guidelines (serves/day) are presented in Table 1. Small but significant increases in vegetables, and small reductions in fruit, grain, fat, and discretionary choice intakes were identified. Despite this, it is interesting to note that significant increases in diet quality were detected as per the ARFS, which is an indicator of increased dietary variety and that intakes were slightly more in line with the Australian Dietary Guidelines over time. Diet quality, measured as per the NRF index was also found to increase from 2001 to 2013, indicating that there was an overall increase in women’s nutrient intakes towards meeting NRVs. On the other hand, small but significant reductions in the MDS were detected from 2001 to 2013, with results demonstrating intakes by Australian women were not well aligned with a Mediterranean style diet, with women on average scoring only 6.6–6.7 out of a possible 17 points (Table 2).

When it came to dietary nitrate intakes, on average Australian women were consuming 65.1 mg/day (95% CI: 64.5 to 65.7) in 2001, with average intakes increasing to 71.7 mg/day (95% CI: 71.0 to 72.4) in 2013 (Table 3). As expected, vegetables were the primary source of dietary nitrate, on average contributing 52.5 mg/day (95% CI: 51.9–53.0; 81% of total nitrate intake) in 2001 and 59.3 mg/day (95% CI: 58.7–60.0; 83% of total nitrate intake) in 2013. Lettuce and spinach were found to be the primary sources of vegetable nitrate at both time points. Lettuce contributed 26% to total vegetable nitrate consumed at both time points. Spinach contributed 11% and 16% to total vegetable nitrate consumed in 2001 and 2013, respectively. Smaller amounts of dietary nitrate were contributed from fruit, unprocessed meats, discretionary choices, and grain foods. Processed meats contributed very little to total dietary nitrate intakes, along with alcohol and dairy foods (Table 3 and Table 4).

With vegetables being the major source of dietary nitrate in this sample, a strong positive correlation was observed between total dietary nitrate intakes and total vegetable intakes. In particular, strong positive correlations were found between total dietary nitrate intakes and green leafy vegetable intake. Moderate positive correlations were observed between total dietary nitrate intakes and diet quality measured by the ARFS and the MDS. However a weak inverse correlation was observed between the NRF index and total dietary nitrate intakes (Table 5).

## 4. Discussion

Based on FFQ data reported within this representative sample of Australian women, in 2001 average dietary nitrate intakes were calculated to be approximately 65.11 mg/day, a level of intake that increased significantly to 71.68 mg/day in 2013. Based on findings from previous literature, it was expected that vegetables would be the primary dietary factor contributing to total dietary nitrate intakes. Our findings indicate that an increase in total vegetable consumption was likely responsible for the significant increase in vegetable nitrate consumption (+6.88 mg/day), which was the primary driver for the overall increase in total nitrate intakes (+6.57 mg/day) over time. This is further supported by our findings which show total dietary nitrate intakes have strong positive correlations with total vegetable intakes (g/day), at both time points. 

Green leafy vegetables including lettuce and spinach have been consistently found to be major dietary sources of nitrate, as they contain relatively large amounts of nitrate (>1000 mg/kg) and are commonly consumed vegetables. Our findings indicated a strong positive correlation between total dietary nitrate intakes and green leafy vegetable (lettuce, spinach, and cabbage) intake (g/day), at both time points. We also found that within this representative sample of mid-aged Australian women, lettuce was the number one source of vegetable nitrate, contributing approximately 13.5 mg/day (26%) at both time points. Although spinach was the second major source of vegetable nitrate at both time points, it was interesting to note that the relative vegetable nitrate contribution from spinach intake jumped from 7.14 mg/day in 2001 to 11.69 mg/day in 2013. This significant increase in nitrate consumption from spinach (+4.56 mg/day), not only in part helps to explain the significant increase in total dietary nitrate intakes, but also represents a major change in how Australian women are consuming vegetables, with women reporting on average a 2.5 g/day increase in spinach intake at 2013 compared with 2001. This increase in spinach consumption could be related to the significant changes in spinach production worldwide, which have occurred since the mid 1990’s as a result of the increased demand for spinach [36]. In the U.S., fresh spinach consumption increased from 0.3 kg/person in 1995 to approximately 1.0 kg/person in 2005, which was linked to the increased availability of convenient, pre-washed, and pre-packaged spinach [36]. Increased availability of convenient sources of a greater variety of vegetables could significantly help to improve overall vegetable intakes in adult Australians, given that current average vegetable intakes are well below the recommended 5 serves/day.

Although Beetroot and celery were consumed in smaller amounts in this cohort, they were the third and fourth major sources of vegetable nitrate, which is primarily due to their high nitrate concentrations (>1000 mg/kg) [13]. Other vegetable nitrate sources, which were consumed in larger amounts, including potato, green beans, pumpkin, broccoli, and carrot, are moderate to low-nitrate containing vegetables (100–420 mg/kg) [13]. Although these vegetables have moderate to low nitrate content, they contribute a considerable amount to the total nitrate consumed in the diet. For example, potatoes contain approximately 150 mg/kg nitrate but contributed to 6–8% of total vegetable nitrate intakes in this cohort. This aligns with results from another Australian cohort where potatoes were shown to be the fifth highest vegetable nitrate source from the diet [21,22]. However, it is important to recognize that the true nitrate content of vegetables can vary dramatically depending on farming, food storage, and food preparation/cooking practices [13,37,38]. This measurement error is a common and widely known limitation of estimating usual dietary intakes from self-reported dietary assessment tools [39], however our use of validated dietary assessment tools such as the DQES and use of comprehensive nitrate databases, which were previously applied to the DQES and tested against objective urinary nitrate levels under controlled conditions, are currently best practice for minimizing measurement error [40].

Consistent with previous literature, we found fruit to be the second major source of dietary nitrate contributing to approximately 6–7% of total dietary nitrate intakes [21]. Nitrate and nitrite salts are commonly used as a preservative in processed meats including ham, bacon, sausages, and salami [34]. Despite this, our findings indicate that processed meats contributed very little (approximately 1%) to overall dietary nitrate intakes. In Australia, the use of nitrate in processed meats is strictly regulated, and there is increasing consumer demand for lower nitrate concentrations in processed meats and/or alternatives [41]. With these two factors in mind, processed meats tend to contribute between 10–90 mg/kg of nitrate, which is considerably lower than most vegetables (60–4000 mg/kg) [34]. Although there is not a lot of world wide data available on processed meat consumption, it is estimated that the proportion of the population which consumes processed meats can vary from 2–65% depending on the country [42]. Globally, Australia is considered to be one of the highest consumers of meat, and on average consumes 24% more red meat than the maximum amount suggested by the Australian Dietary Guidelines (ADG). Despite this, our sample reported consuming approximately 17 g/day of processed meats, which is equivalent to approximately 0.5 serves/day, and although exceeds recommended intakes, explains the overall low nitrate contribution from processed meats [42].

According to the ADG, Healthy Australian adults should be aiming to consume 5 serves of vegetables/day as a primary means of reducing risk of developing chronic conditions including cardiovascular disease, diabetes, and certain cancers. We found in our representative sample of mid-aged Australian Women, that less than 2% of the population were consuming 5 or more serves of vegetables per day, and the average daily intakes of vegetables for our sample was approximately 2.3 serves/day, a level of intake which contributes around 50–60 mg/day of dietary nitrate. With this in mind, high quality evidence from a systematic review and meta-analysis of experimental trials indicates that dietary nitrate intakes of around 130 mg/day have potent cardioprotective effects [20], thus in theory, if women could double their vegetable consumption to be aligned with the ADG, then this would help women to achieve higher, potentially therapeutic, dietary nitrate intakes, especially if high nitrate containing vegetables such as green leafy vegetables were emphasized. Although, experimental data has suggested a dose–effect relationship between dietary nitrate and CVD risk factors including blood pressure [20,43], this finding is not consistent across the literature indicating a potential therapeutic threshold for dietary nitrate intakes. Although this hypothesis requires further investigation, it is possible that higher dietary nitrate intakes don’t equate to better health outcomes, rather moderate nitrate intakes may be sufficient.

Generally, higher vegetable intakes are associated with an overall higher diet quality, in particular the ARFS is known to score generously based on total vegetable intakes and variety, and we found the ARFS to be moderately positively correlated with total dietary nitrate intakes. However, in our sample of Australian women, overall ARFS scores were on average 32–33 out of a possible maximum score of 74, again reinforcing the notion that mid-aged Australian women do not consume diets in line with the ADG. 

Our data also indicates that the diets of mid-aged Australian women are not well aligned with a Mediterranean style of eating, scoring on average only 6.6–6.7 out of a possible maximum score of 17. In particular our data highlights low MDS scores for legume and fish consumption, but despite overall low MDS in this sample, the MDS was also found to be moderately positively correlated with total dietary nitrate intakes.

Contrary to the ARFS and MDS, we found weak to moderate inverse correlations between the NRFI with total nitrate consumption. These extreme differences in correlations could be linked to the fact that the ARFS and MDS are food focused diet quality scores, while the NRFI has a focus on nutrient intakes in reference to national NRVs. Based on the NRFI, it was interesting to note that the score increased from 78.37 in 2001 to 86.78 in 2013, despite the fact that many nutrient rich foods in particular fruit and grain consumption were reduced during this timeframe. For example, on average women reported reduced fruit consumption by 0.25 serves/day, which over one week is equivalent to 1.75 serves of fruit. Also, women reported reduced grain consumption by 0.6 serves/day, a reduction in grain intake which is equivalent to over 4 slices of bread per week. However, such reductions in fruit could account for reductions in total sugar consumption (−5.87 g/day) (which was considered to be a negative nutrient) and reductions in grain consumption, in particular processed grains, could account for a reduction in sodium (−249.35 mg/day) (also considered a negative nutrient in the NRFI). Although, a major limitation of the DQES is that in general core food items (which have favorable nutrient profiles) are better represented than non-core food items (which have poor nutrient profiles). For example, the list of vegetable, fruit, grain, and dairy foods are reasonably extensive within the DQES, while few discretionary food items including processed meats, takeaway, and convenience food items are represented within the DQES, and could represent another explanation for the inverse association observed between the NRFI and total nitrate intakes. 

In recent years there has been an increasing emphasis placed on understanding overall diet quality and dietary patterns as part of nutrition research and health. This is because it is important to consider all dietary nutrients and components, as their effects can be interdependent, and explains why better health outcomes are frequently observed in studies using whole food intakes compared with isolated nutrient supplements. In fact, it is thought that the biochemistry of nitrate is favored to produce NO in the human body when in the presence of other beneficial dietary components including polyphenols and vitamin C [24,44,45].

In addition, the microbiome is a rapidly growing area of investigation. Bacteria in the oral cavity are known to have a key role in facilitating the nitrate-nitrite-NO pathway, thus it is reasonable to hypothesize that the gut microbiome may also have a role in a mediating the nitrate-nitrite-NO pathway. Furthermore, Gilbert et al. suggest that the gut microbiome and diet represents an avenue of research which could yield new therapeutic strategies for conditions in which the gut microbiome and its metabolites have been shown to be disease-causative/preventative [46]. So, although this hypothesis requires substantially more research investigation, again it highlights the importance of overall diet quality when considering the health promoting effects of dietary nitrate, particularly as gut microbiome diversity is promoted by intakes of a variety of fiber rich plant based foods including vegetables, fruit, nuts, and wholegrains, components which are important for diet quality [46].

This analysis is based on the only population data available in Australia, in which diet information has been collected using the same validated FFQ within the exact same individuals followed up over 12 years, allowing us to make clear comparisons in dietary changes over time. In addition, the 12-year follow-up period (2001–2013) for this representative sample of mid-aged Australian women represents a stage of significant lifestyle changes, and increasing risk of age related disease and poor health. Thus, represents an opportune time to explore changes in dietary habits and diet quality, given diet is one of the primarily modifiable risk factors in health [47]. 

Dietary nitrate intakes have been estimated previously within an Australian cohort (Calcium Intake Fracture Outcome Study) of 1226 women aged 70–85 years old in 1998. Mean vegetable nitrate intakes within this cohort were approximately 67 mg/day, a level of intake approximately 7 mg/day higher than the vegetable nitrate values calculated in our sample of women aged 62–67 in 2013 [21]. These findings are despite the fact that we used the same vegetable nitrate database and the same FFQ (the DQES) [13,28], however may indicate that vegetable nitrate intakes continue to increase with age. On the other hand, it is also important to recognize the significant changes in food trends and the significant increase in the variety of food choices which have occurred in Australia between 1998 and 2013. Further, it is likely the intakes using the DQES were better able to capture whole dietary patterns and vegetable intakes in 1998, compared with dietary patterns and vegetable intakes in 2013. For example, vegetable items including sweet potato, rocket, kale, and other green leafy vegetables such as bok choy are not vegetable items included as part of the DQES, but their consumption is increasing within an Australian context. In fact, the volume of sweet potato sales in Australia increased by a significant 21% in 2016 compared with the previous year [48]. Furthermore, in 2017 volume sales of Asian green vegetables jumped by 22% compared with sales in 2016 [49]. Therefore, due to the DQES not including these vegetables (which are moderate to high nitrate containing vegetables), the vegetable data collected in 2013 as part of the DQES, may not be capturing true vegetable intakes compared with intakes collected 1998 using the DQES, a period when less vegetable options were available and commonly consumed. Furthermore, this may provide another explanation for why estimated vegetable nitrate intake in our sample were lower than those calculated in another sample of Australian women, however given the 10+ year age difference between the two cohorts, it is likely that women aged 70 or more are less likely to make changes to the types of vegetables they consume based on trends.

## 5. Conclusions

Dietary nitrate increasingly appears to have a protective role in health, especially vascular health as nitrate can be reduced in the body to produce nitric oxide. Vegetables are one of the primary sources of dietary nitrate, and dietary nitrate intakes were found to be significantly greater in mid-aged Australian women consuming better quality diets. Although on average we found dietary nitrate intakes to be lower than doses shown to have therapeutic effects, therapeutic doses of dietary nitrate could be obtained if women increased their vegetable consumption to be consistent with the Australian Dietary Guidelines. Although there is emerging evidence indicating that higher habitual dietary nitrate intakes are associated with reduced morbidity and mortality. It is important that future work in this area considers how dietary nitrate within the context of overall diet quality can facilitate health. This work will ensure the health messages of this growing area of research are translatable to positive public health messages. 

## Figures and Tables

**Table 1:** Summary and comparison of dietary intakes in 2001 and 2013: Food group serves per day, based on the Australian Dietary Guidelines.

	FFQ Data 2001 (Women 50–55 Year)		FFQ Data 2013 (Women 62–67 Year)		Change over Time	
Dietary Component	Median (IQR)	Mean (95% CI)	Median (IQR)	Mean (95% CI)	Median (IQR)	Mean (95% CI)
Vegetable serves/day ^‡^	2.11 (1.26)	2.27 (2.24 to 2.29)	2.22 (1.26)	2.36 (2.34 to 2.38)	+0.10 (1.15)	+0.09 (0.07 to 0.12)
Fruit serves/day ^‡^	1.79 (1.63)	1.98 (1.95 to 2.01)	1.59 (1.38)	1.71 (1.69 to 1.74)	−0.18 (1.33)	−0.27 (−0.29 to 0.24)
Grain serves/day ^‡^	3.52 (2.09)	3.74 (3.70 to 3.78)	2.95 (1.97)	3.14 (3.10 to 3.18)	−0.53 (2.12)	−0.60 (−0.64 to −0.56)
Dairy serves/day	1.70 (1.04)	1.77 (1.75 to 1.78)	1.65 (0.98)	1.73 (1.71 to 1.74)	0 (0.95)	−0.04 (−0.06 to −0.02)
Meat serves/day ^†^	1.71 (1.23)	1.99 (1.96 to 2.03)	1.75 (1.18)	1.99 (1.96 to 2.01)	+0.04 (1.20)	−0.01 (−0.05 to 0.02)
Discretionary Choices serves/day *^,†^	1.66 (1.53)	2.05 (2.01 to 2.08)	1.62 (1.51)	1.96 (1.93 to 1.99)	−0.05 (1.35)	−0.09 (−0.12 to −0.05)
Fat g/day ^‡^	14.0 (21.0)	12.91 (12.68 to 13.13)	14.0 (10.5)	10.84 (10.66 to 11.03)	0 (10.5)	−2.06 (−2.29 to −1.83)

Serves calculated as per the Australian Dietary Guidelines. One vegetable serve = 75 g; One fruit serve = 150 g; One grain serve = 30–120 g; One dairy serve = 40–250 g; One meat serve = 65–100 g; One discretionary choice serve = 25–75 g. ^‡^ *p* value for change over time was <0.0001. * Discretionary choices, includes all processed meats: ham, bacon, sausages, and salami as per the Australian Dietary Guidelines, plus food items including fats and oils, sugar, biscuits, cakes, pastries, takeaway (hamburgers, hot chips), crisps, and ice cream. ^†^ *p* value for change over time was <0.001.

**Table 2:** Summary and comparison of Diet Quality in 2001 and 2013: Australian Recommended Food Score, Nutrient Rich Foods Index and Mediterranean Diet Score.

	FFQ Data 2001 (Women 50–55 Year)		FFQ Data 2013 (Women 62–67 Year)		Change over Time	
Dietary Component	Median (IQR)	Mean (95% CI)	Median (IQR)	Mean (95% CI)	Median (IQR)	Mean (95% CI)
Total ARFS ^‡^ (max 74)	33 (11)	32.37 (32.18 to 32.56)	33 (12)	32.83 (32.64 to 33.02)	0 (9)	+0.46 (0.29 to 0.62)
Total NRF Index ^‡^	77.58 (33.57)	78.37 (77.80 to 78.94)	85.78 (36.1)	86.78 (86.18 to 87.37)	+7.68 (32.02)	+8.41 (7.83 to 8.98)
Total MDS ^‡^ (max 17)	7 (2)	6.72 (6.55 to 6.76)	7 (3)	6.60 (6.56 to 6.63)	0 (2)	−0.12 (−0.16 to −0.08)

ARF: Australian Recommended Food Score; NRF Index: Nutrient Rich Foods Index; MDS: Mediterranean Diet Score. ^‡^ *p* value for change over time was <0.0001.

**Table 3:** Summary and comparison of dietary intakes in 2001 and 2013: Total dietary nitrate consumption and nitrate contribution by food group.

	FFQ Data 2001 (Women 50–55 Year)		FFQ Data 2013 (Women 62–67 Year)		Change over Time	
Dietary Component (mg/day)	Median (IQR)	Mean (95% CI)	Median (IQR)	Mean (95% CI)	Median (IQR)	Mean (95% CI)
Total dietary Nitrate ^‡^	60.90 (33.21)	65.11 (64.51 to 65.72)	67.14 (36.55)	71.68 (71.01 to 72.35)	+5.52 (33.57)	+6.57 (5.90 to 7.23)
Total Vegetable Nitrate ^‡^	48.50 (30.31)	52.45 (51.89 to 53.01)	54.75 (34.58)	59.34 (58.70 to 60.00)	+5.49 (31.08)	+6.88 (6.26 to 7.51)
Total Fruit Nitrate ^†^	4.00 (4.05)	4.47 (4.41 to 4.54)	4.13 (3.69)	4.36 (4.31 to 4.42)	−0.07 (3.15)	−0.11 (−0.17 to −0.05)
Total Processed Meat Nitrate	0.49 (0.70)	0.75 (0.72 to 0.77)	0.49 (0.7)	0.71 (0.70 to 0.73)	0 (0.6)	−0.03 (−0.06 to −0.01)
Total Grain Nitrate ^‡^	1.84 (1.20)	2.10 (2.07 to 2.12)	1.75 (1.43)	2.03 (2.00 to 2.06)	−0.10 (1.37)	−0.07 (−0.10 to −0.03)
Total Meat Nitrate	1.87 (1.55)	2.29 (2.25 to 2.34)	1.85 (1.41)	2.20 (2.16 to 2.23)	−0.0006 (1.4)	−0.10 (−0.14 to −0.05)
Total Dairy Nitrate ^‡^	0.13 (0.08)	0.13 (0.13 to 0.14)	0.13 (0.08)	0.14 (0.14 to 0.14)	+0.001 (0.44)	+0.002 (0.001 to 0.004)
Total Alcohol Nitrate ^‡^	0.05 (0.19)	0.15 (0.14 to 0.15)	0.04 (0.18)	0.13 (0.12 to 0.13)	0 (0.06)	−0.02 (−0.024 to −0.016)
Total Discretionary Food Nitrate *	2.09 (2.29)	2.83 (2.77 to 2.89)	2.08 (2.44)	2.83 (2.77 to 2.89)	−0.03 (2.22)	+0.001 (−0.07 to 0.06)

Food groups determined as per the Australian Dietary Guidelines ^‡^ *p* value for change over time was <0.0001; ^†^ *p* value for change over time was <0.001; * *p* value for change over time was <0.05.

**Table 4:** Summary and comparison of dietary intakes in 2001 and 2013: Vegetable dietary nitrate consumption by nitrate contributing vegetables.

	FFQ Data 2001 (Women 50–55 Year)		FFQ Data 2013 (Women 62–67 Year)		Change over Time	
Dietary Component (mg/day)	Median (IQR)	Mean (95% CI)	Median (IQR)	Mean (95% CI)	Median (IQR)	Mean (95% CI)
Total Vegetable Nitrate ^‡^	48.50 (30.31)	52.45 (51.89 to 53.01)	54.75 (34.58)	59.34 (58.70 to 60.00)	+5.49 (31.08)	+6.88 (6.26 to 7.51)
Lettuce Nitrate	11.96 (11.80)	13.44 (13.23 to 13.65)	12.13 (12.31)	13.58 (13.36 to 13.79)	0 (11.29)	+0.14 (−0.10 to 0.38)
Spinach Nitrate ^‡^	2.88 (7.93)	7.14 (6.89 to 7.38)	6.49 (14.78)	11.69 (11.35 to 12.04)	+1.44 (9.37)	+4.56 (4.21 to 4.91)
Beetroot Nitrate ^‡^	2.86 (7.14)	5.67 (5.52 to 5.83)	3.57 (7.43)	6.04 (5.88 to 6.20)	0 (5.58)	+0.36 (0.18 to 0.54)
Celery Nitrate ^‡^	2.38 (5.21)	3.85 (3.76 to 3.94)	3.09 (5.94)	4.53 (4.43 to 4.63)	+0.24 (4.04)	+0.68 (0.57 to 0.78)
Potato Nitrate ^‡^	3.28 (4.22)	4.10 (4.01 to 4.17)	2.59 (3.94)	3.45 (3.37 to 3.52)	−0.40 (3.62)	−0.65 (−0.73 to −0.56)
Green Beans Nitrate ^‡^	2.38 (3.30)	2.97 (2.92 to 3.03)	2.89 (3.57)	3.37 (3.31 to 3.42)	+0.31 (3.00)	+0.39 (0.33 to 0.46)
Pumpkin Nitrate ^‡^	2.32 (3.83)	3.38 (3.30 to 3.45)	2.67 (3.73)	3.56 (3.49 to 3.63)	+0.18 (2.99)	+0.18 (0.10 to 0.26)
Broccoli Nitrate ^‡^	2.21 (3.06)	2.73 (2.68 to 2.78)	3.00 (3.53)	3.44 (3.38 to 3.50)	+0.48 (2.86)	+0.71 (0.65 to 0.77)
Cabbage Nitrate	1.67 (3.45)	2.93 (2.85 to 3.01)	1.71 (3.67)	2.99 (2.91 to 3.07)	0 (2.65)	+0.06 (−0.03 to 0.15)
Carrot Nitrate ^‡^	1.73 (1.80)	2.04 (2.00 to 2.07)	1.81 (1.78)	2.12 (2.08 to 2.15)	+0.09 (1.62)	+0.08 (0.04 to 0.11)
Cauliflower Nitrate ^‡^	0.86 (1.54)	1.34 (1.31 to 1.37)	1.16 (2.00)	1.69 (1.65 to 1.72)	+0.17 (1.44)	+0.35 (0.31 to 0.39)
Other Vegetable Nitrate *	2.67 (1.84)	2.67 (2.84 to 2.90)	2.69 (1.84)	2.89 (2.86 to 2.93)	+0.03 (1.77)	+0.03 (−0.06 to 0.01)

^‡^*p* value for change over time was <0.0001. * Other Vegetables Include: Tomato, Capsicum, Cucumber, Peas, Bean sprouts, Baked beans, Onion, Garlic, and Mushroom.

**Table 5:** Dietary components and diet quality correlations with total dietary nitrate.

	FFQ Data 2001 (Women 50–55 Year)		FFQ Data 2013 (Women 62–67 Year)	
Dietary Component	Dietary Nitrate, *r*	*p*-Value	Dietary Nitrate, *r*	*p*-Value
Vegetable intake (g/day)	0.79	<0.0001	0.78	<0.0001
Green leafy vegetable intake (g/day) *	0.81	<0.0001	0.83	<0.0001
ARFS	0.36	<0.0001	0.34	<0.0001
MDS	0.33	<0.0001	0.29	<0.0001
NRF Index	−0.18	<0.0001	−0.14	<0.0001

* Green leafy vegetables include lettuce, spinach, and cabbage ARF: Australian Recommended Food Score; NRF Index: Nutrient Rich Foods Index; MDS: Mediterranean Diet Score.

## Supplementary Materials

The following are available online at http://www.mdpi.com/2072-6643/10/8/1005/s1, Table S1: Criteria for calculating Nutrient Rich Foods Index (NRFI), Table S2: Criteria for Calculating the Australian Recommended Food Score (ARFS), Table S3: Criteria for Calculating the Mediterranean Diet Score (MDS).
