# Supplementary material for: Dietary Nitrate and Diet Quality: An Examination of Changing Dietary Intakes within a Representative Sample of Australian Women
Source: Nutrients. 2018 Aug 1;10(8):1005. doi: 10.3390/nu10081005 (PMC6116056; doi:10.3390/nu10081005)
Supplement: Supplementary file 1 [file nutrients-10-01005-s001.pdf]

nutrients

MDPI

**Supplementary Material**

**Table S1: Criteria for calculating Nutrient Rich Foods Index (NRFI)**

| Positive Nutrient Rich Foods Component | Nutrient Reference Value | Minimum Percentage Adherence | Maximum Percentage Adherence |
|---|---|---|---|
| Protein | RDI: 46 g/d | 0 | Capped at 100 |
| Fiber | AI: 25 g/d | 0 | Capped at 100 |
| Vitamin A | RDI: 700 µg/d | 0 | Capped at 100 |
| Vitamin C | RDI: 45 mg/d | 0 | Capped at 100 |
| Vitamin E | AI: 7 mg/d | 0 | Capped at 100 |
| Calcium | RDI: 1300 mg/d | 0 | Capped at 100 |
| Iron | RDI: 8 mg/d | 0 | Capped at 100 |
| Potassium | RDI: 2800 mg/d | 0 | Capped at 100 |
| Magnesium | RDI: 320 mg/d | 0 | Capped at 100 |
| **Negative Nutrient Rich Foods Component** | **Nutrient Reference Value** | **Minimum Percentage Adherence** | **Maximum Percentage Adherence** |
| Sodium | UL: 2300 mg/d | 0 | May exceed 100 |
| Saturated Fat | Daily Intake Guide: 24 g/d | 0 | May exceed 100 |
| Sugar | Daily Intake Guide: 90 g/d | 0 | May exceed 100 |

Diet Nutrient Density Score = (Sum of percentage adherence to positive nutrients) – (Sum of percentage adherence to negative nutrients)

NRFI = (Diet Nutrient Density Score, standardized per 1000 kJ of energy intake)

RDI: Recommended Daily Intake; AI: Adequate Intake; UL: Upper Limit

**Table S2. Criteria for calculating the Australian Recommended Food Score (ARFS)**

| ARFS sub-group | DQSE item | Level of Intake | Scoring |
|---|---|---|---|
| Vegetables (max score=22) | How many serves of vegetables/d | > 4 serves /d | 1 |
| | | ≤ 4 serves/d | 0 |
| | Potato | >1 serve/wk | 1 |
| | | ≤1 serve/wk | 0 |
| | Tomato sauce | >1 serve/wk | 1 |
| | | ≤1 serve/wk | 0 |
| | Fresh/tinned tomato | >1 serve/wk | 1 |
| | | ≤1 serve/wk | 0 |
| | Capsicum | >1 serve/wk | 1 |
| | | ≤1 serve/wk | 0 |
| | Lettuce/salad greens | >1 serve/wk | 1 |
| | | ≤1 serve/wk | 0 |
| | Cucumber | >1 serve/wk | 1 |
| | | ≤1 serve/wk | 0 |
| | Celery | >1 serve/wk | 1 |
| | | ≤1 serve/wk | 0 |
| | Beetroot | >1 serve/wk | 1 |
| | | ≤1 serve/wk | 0 |
| | Carrot | >1 serve/wk | 1 |
| | | ≤1 serve/wk | 0 |
| | Cabbage/ Brussel sprouts | >1 serve/wk | 1 |
| | | ≤1 serve/wk | 0 |
| | Cauliflower | >1 serve/wk | 1 |
| | | ≤1 serve/wk | 0 |
| | Broccoli | >1 serve/wk | 1 |
| | | ≤1 serve/wk | 0 |
| | Spinach | >1 serve/wk | 1 |
| | | ≤1 serve/wk | 0 |
| | Peas | >1 serve/wk | 1 |
| | | ≤1 serve/wk | 0 |
| | Green Beans | >1 serve/wk | 1 |
| | | ≤1 serve/wk | 0 |
| | Beans/Bean spouts | >1 serve/wk | 1 |
| | | ≤1 serve/wk | 0 |
| | Pumpkin | >1 serve/wk | 1 |
| | | ≤1 serve/wk | 0 |
| | Onions | >1 serve/wk | 1 |
| | | ≤1 serve/wk | 0 |
| | Garlic | >1 serve/wk | 1 |
| | | ≤1 serve/wk | 0 |
| | Mushrooms | >1 serve/wk | 1 |
| | | ≤1 serve/wk | 0 |
| | Zucchini | >1 serve/wk | 1 |
| | | ≤1 serve/wk | 0 |
| Fruit (max score=14) | How many serves of fruit/d | > 2 serves /d | 1 |
| | | ≤ 2 serves/d | 0 |
| | Fruit Juice | >1 serve/wk | 1 |
| | | ≤1 serve/wk | 0 |
| | Tinned/frozen fruit | >1 serve/wk | 1 |
| | | ≤1 serve/wk | 0 |
| | Oranges | >1 serve/wk | 1 |
| | | ≤1 serve/wk | 0 |

| | | | |
|---|---|---|---|
| | Apples | >1 serve/wk | 1 |
| | | ≤1 serve/wk | 0 |
| | Pears | >1 serve/wk | 1 |
| | | ≤1 serve/wk | 0 |
| | Banana | >1 serve/wk | 1 |
| | | ≤1 serve/wk | 0 |
| | Melon | >1 serve/wk | 1 |
| | | ≤1 serve/wk | 0 |
| | Pineapple | >1 serve/wk | 1 |
| | | ≤1 serve/wk | 0 |
| | Strawberries | >1 serve/wk | 1 |
| | | ≤1 serve/wk | 0 |
| | Apricots | >1 serve/wk | 1 |
| | | ≤1 serve/wk | 0 |
| | Peaches | >1 serve/wk | 1 |
| | | ≤1 serve/wk | 0 |
| | Mango | >1 serve/wk | 1 |
| | | ≤1 serve/wk | 0 |
| | Avocado | >1 serve/wk | 1 |
| | | ≤1 serve/wk | 0 |
| Grains (max score=14) | Consume high fiber white bread | Yes | 1 |
| | | No | 0 |
| | Consume wholemeal bread | Yes | 1 |
| | | No | 0 |
| | Consume rye bread | Yes | 1 |
| | | No | 0 |
| | Consume multi-grain bread | Yes | 1 |
| | | No | 0 |
| | How many slices of bread/d | ≥4 slices/d | 1 |
| | | <4 slices/d | 0 |
| | All bran | >1 serve/wk | 1 |
| | | ≤1 serve/wk | 0 |
| | Sultana bran | >1 serve/wk | 1 |
| | | ≤1 serve/wk | 0 |
| | Weetbix | >1 serve/wk | 1 |
| | | ≤1 serve/wk | 0 |
| | Rice | >1 serve/wk | 1 |
| | | ≤1 serve/wk | 0 |
| | Pasta/Noodles | >1 serve/wk | 1 |
| | | ≤1 serve/wk | 0 |
| | Vegemite | >1 serve/wk | 1 |
| | | ≤1 serve/wk | 0 |
| | Cornflakes | >1 serve/wk | 1 |
| | | ≤1 serve/wk | 0 |
| | Porridge | >1 serve/wk | 1 |
| | | ≤1 serve/wk | 0 |
| | Muesli | >1 serve/wk | 1 |
| | | ≤1 serve/wk | 0 |
| Dairy (max score=7) | Consume reduced fat or skim milk | Yes | 1 |
| | | No | 0 |
| | Consume soy milk | Yes | 1 |
| | | No | 0 |
| | Consume low fat cheese | Yes | 1 |
| | | No | 0 |
| | How much milk/d | >500 ml/d | 1 |
| | | ≤500 ml/d | 0 |

| | | | |
|---|---|---|---|
| | Cheese | <1 serve/wk | 1 |
| | | ≥1 serve/wk | 0 |
| | Ice-cream | <1 serve/wk | 1 |
| | | ≥1 serve/wk | 0 |
| | Yoghurt | >1 serve/wk | 1 |
| | | ≤1 serve/wk | 0 |
| Nuts/beans/soy/egg (max score=7) | Nuts | >1 serve/wk | 1 |
| | | ≤1 serve/wk | 0 |
| | Peanut Butter | >1 serve/wk | 1 |
| | | ≤1 serve/wk | 0 |
| | Baked beans | >1 serve/wk | 1 |
| | | ≤1 serve/wk | 0 |
| | Soy/tofu | >1 serve/wk | 1 |
| | | ≤1 serve/wk | 0 |
| | Soy milk | Yes | 1 |
| | | No | 0 |
| | Other Beans | >1 serve/wk | 1 |
| | | ≤1 serve/wk | 0 |
| | Eggs | 1-5 eggs/wk | 1 |
| | | <1 or >5 eggs/wk | 0 |
| Meat (max score=5) | Beef | 1-4 serves/wk | 1 |
| | | <1 or >4 serves/wk | 0 |
| | Veal | 1-4 serves/wk | 1 |
| | | <1 or >4 serves/wk | 0 |
| | Lamb | 1-4 serves/wk | 1 |
| | | <1 or >4 serves/wk | 0 |
| | Pork | 1-4 serves/wk | 1 |
| | | <1 or >4 serves/wk | 0 |
| | Chicken | 1-4 serves/wk | 1 |
| | | <1 or >4 serves/wk | 0 |
| Fish (max score=2) | Fish | 1-4 serves/wk | 1 |
| | | <1 or >4 serves/wk | 0 |
| | Tinned Fish | 1-4 serves/wk | 1 |
| | | <1 or >4 serves/wk | 0 |
| Fat (max score=1) | Consume Poly-unsaturated, Mono-unsaturated spread or do not use spread | Yes | 1 |
| | | No | 0 |
| Alcohol (max score=2) | How often is alcohol consumed | 1-4 days/wk | 1 |
| | | <1 or >4 days/wk | 0 |
| | How many when alcohol is consumed | 1-2 standard drinks | 1 |
| | | <1 or >2 standard drinks | 0 |

**Table S3. Criteria for calculating the Mediterranean Diet Score (MDS)**

| Food Group | Level of Intake | Scoring |
|---|---|---|
| Vegetables | >250 gm/d | 2 |
| | 100-250 gm/d | 1 |
| | <100 gm/d | 0 |
| Fruit and Nuts | >300 gm/d | 2 |
| | 150-300 gm/d | 1 |
| | <150 gm/d | 0 |
| Legumes | >140 gm/d | 2 |
| | 70-140 gm/d | 1 |
| | <70 gm/d | 0 |
| Grains | >195 gm/d | 2 |
| | 130-195 gm/d | 1 |
| | <130 gm/d | 0 |
| Meat | >120 gm/d | 0 |
| | 80-120 gm/d | 1 |
| | <80 gm/d | 2 |
| Fish | >250 gm/d | 2 |
| | 100-250 gm/d | 1 |
| | <100 gm/d | 0 |
| Dairy | >270 gm/d | 1 |
| | 180-270 gm/d | 2 |
| | <180 gm/d | 0 |
| Alcohol | >24 gm/d | 0 |
| | 12-24 gm/d | 2 |
| | <12 gm/d | 1 |
| Use Mono-unsaturated fat | Yes | 1 |
| | No | 0 |

**Table S4. Summary of Diet Quality Scores their Component Scores and additional key Macro and Mico-nutrient intake data**

| | FFQ data 2001 (Women 50-55 yr) | | FFQ data 2013 (Women 62-67 yr) | | Change over time | |
|---|---|---|---|---|---|---|
| **ARFS and sub-scale component scores** | **Median (IQR)** | **Mean (95% CI)** | **Median (IQR)** | **Mean (95% CI)** | **Median (IQR)** | **Mean change** |
| Total ARFS (max 74)‡ | 33 (11) | 32.4 (32.2-32.6) | 33 (12) | 32.8 (32.6-33.0) | 0 (9) | +0.5 |
| ARFS vegetable sub-scale (max 22)† | 14 (6) | 13.7 (13.6-13.8) | 14 (6) | 13.8 (13.7-13.9) | 0 (5) | +0.1 |
| ARFS fruit sub-scale (max 14) | 6 (5) | 5.6 (5.5-5.7) | 6 (5) | 5.7 (5.6-5.7) | 0 (4) | +0.03 |
| ARFS grain Sub-score (max 14)‡ | 4 (2) | 4.1 (4.1-4.1) | 4 (2) | 3.8 (3.8-3.9) | 0 (3) | -0.3 |
| ARFS dairy Sub-scale (max 7)‡ | 2 (2) | 2.1 (2.1-2.1) | 2 (1) | 2.2 (2.2-2.2) | 0 (2) | +0.01 |
| ARFS nuts/beans/soy/eggs Sub-scale (max 7)‡ | 2 (2) | 2.0 (2.0-2.0) | 2 (2) | 2.3 (2.2-2.3) | 0 (1) | +0.2 |
| ARFS meat Sub-scale (max 5)‡ | 2 (1) | 2.4 (2.4-2.4) | 3 (1) | 2.5 (2.4-2.5) | 0 (2) | +0.09 |
| ARFS fish Sub-scale (max 2)‡ | 1 (2) | 0.9 (0.8-0.9) | 1 (2) | 1.0 (1.0-1.0) | 0 (1) | +0.1 |
| ARFS alcohol Sub-scale (max 2)‡ | 1 (2) | 0.9 (0.2-0.9) | 1 (2) | 1.1 (1.1-1.1) | 0 (1) | +0.2 |
| **MDS and sub-score component scores** | **Median (IQR)** | **Mean (95% CI)** | **Median (IQR)** | **Mean (95% CI)** | **Median (IQR)** | **Mean change** |
| Total MDS (max 17)‡ | 7 (2) | 6.7 (6.6-6.8) | 7 (3) | 6.6 (6.6-6.6) | 0 (2) | -0.12 |
| MDS Vegetable component (max 2)‡ | 1 (0) | 0.9 (0.9-1.0) | 1 (0) | 1.0 (1.0-1.0) | 0 (0) | +0.03 |
| MDS Fruit and Nuts component (max 2)‡ | 1 (1) | 1.2 (1.2-1.2) | 1 (2) | 1.1 (1.1-1.1) | 0 (1) | -0.1 |
| MDS Legumes component (max 2)* | 0 (0) | 0.0 (0.0-0.0) | 0 (0) | 0.0 (0.0-0.0) | 0 (0) | +0.003 |
| MDS Grains (max 2)‡ | 1 (1) | 1.2 (1.2-1.3) | 1 (2) | 1.1 (1.1-1.1) | 0 (1) | -0.2 |

| | | | | | | |
|---|---|---|---|---|---|---|
| MDS Meat (max 2)‡ | 1 (2) | 1.2 (1.2-1.2) | 2 (1) | 1.4 (1.3-1.4) | 0 (1) | +0.1 |
| MDS Fish (max 2) | 0 (0) | 0.0 (0.0-0.0) | 0 (0) | 0.0 (0.0-0.0) | 0 (0) | 0.0 |
| MDS Dairy (max 2)† | 0 (1) | 0.4 (0.4-0.4) | 0 (1) | 0.4 (0.4-0.4) | 0 (0) | -0.02 |
| MDS Alcohol (max 2)‡ | 2 (1) | 1.6 (1.6-1.6) | 2 (1) | 1.6 (1.6-1.6) | 0 (0) | +0.03 |
| MDS fat (max 1)‡ | 0 (0) | 0.1 (0.1-0.1) | 0 (0) | 0.1 (0.1-0.1) | 0 (0) | -0.02 |
| **NFR Index and Components** | **Median (IQR)** | **Mean (95% CI)** | **Median (IQR)** | **Mean (95% CI)** | **Median (IQR)** | **Mean change** |
| Total NRF Index‡ | 77.6 (33.5) | 78.4 (77.8-78.9) | 85.8 (36.0) | 86.8 (86.2-87.4) | 7.7 (32) | +8.4 |
| % adherence to protein RDI‡ | 100 (0) | 98.9 (98.8- 99.0) | 100 (0) | 98.5 (98.4-98.6) | 0 (0) | -0.4 |
| % adherence to fiber AI† | 76.5 (39.0) | 75.2 (74.7- 75.6) | 74.9 (36.8) | 73.9 (73.4-74.3) | 0 (25.9) | -1.3 |
| % adherence to vitamin A RDI | 99.2 (24.1) | 86.7 (86.3- 87.1) | 100 (23.5) | 87.1 (86.7-87.4) | 0 (15.3) | +0.3 |
| % adherence to vitamin C RDI | 100 (0) | 98.9 (98.8-99.0) | 100 (0) | 98.1 (98.7-99.0) | 0 (0) | -0.1 |
| % adherence to vitamin E AI‡ | 75.1 (39.2) | 74.2 (73.7- 74.6) | 76.9 (39.7) | 75.5 (75.0 – 75.9) | 0 (25.9) | +1.3 |
| % adherence to calcium RDI† | 63.8 (30.6) | 64.7 (64.2- 65.1) | 62.1 (28.8) | 63.3 (62.9-63.7) | -0.6 (26) | -1.4 |
| % adherence to iron RDI‡ | 100 (0) | 95.5 (95.3- 95.7) | 100 (2.6) | 94.4 (94.2-94.7) | 0 (0) | -1.1 |
| % adherence to potassium RDI‡ | 90.3 (26.6) | 84.9 (84.5- 85.3) | 87.8 (28.6) | 83.5 (83.2-83.9) | 0 (18.7) | -1.4 |
| % adherence to magnesium RDI* | 78.4 (36.2) | 77.4 (76.9- 77.8) | 77.6 (35.2) | 76.4 (76.0-76.8) | 0 (23.8) | -1.0 |
| % adherence to sodium UL‡ | 86.9 (41.5) | 93.0 (92.2-93.9) | 76.7 (41.8) | 82.2 (81.4-83.0) | -9.3 (37) | -10.8 |
| % adherence to saturated fat daily intake guide | 95.1 (59.4) | 104.6 (103.5-105.7) | 94.6 (54.4) | 102.3 (101.2-103.3) | -0.3 (51.9) | -2.3 |

| % adherence to sugar daily intake guide‡ | 85.8 (44.5) | 89.9 (89.1- 90.6) | 79.2 (40.5) | 83.3 (82.6-84.0) | -5.3 (39.8) | -6.5 |
|---|---|---|---|---|---|---|
| **Nutrients** | **Median (IQR)** | **Mean (95% CI)** | **Median (IQR)** | **Mean (95% CI)** | **Median (IQR)** | **Mean change** |
| Energy (kJ/d)‡ | 6331.5 (2736.8) | 6687.9 (6634.8-6741.0) | 5862.1 (2631.2) | 6197.8 (6146.9-6248.6) | -394.5 (2465.4) | -490.1 |
| Protein (g/d)‡ | 76.7 (34.0) | 82.3 (81.6-83.1) | 72.6 (32.3) | 77.3 (76.6-78.0) | -3.3 (31.2) | -5.0 |
| Fibre (g/d)‡ | 19.1 (9.8) | 20.4 (20.2-20.5) | 18.7 (9.2) | 19.7 (19.6-19.9) | -0.4 (9.0) | -0.6 |
| Retinol Equivalents (μg/d) | 694.5 (357.7) | 732.1 (725.9-738.3) | 686.5 (342.7) | 725.9 (720.1-731.8) | +3.2 (334.2) | -6.2 |
| Vitamin C (mg/d)‡ | 103.9 (74.7) | 117.4 (116.0-118.8) | 96.2 (59.8) | 105.4 (104.3-106.5) | -6.8 (65.6) | -12.0 |
| Vitamin E (mg/d)‡ | 5.3 (2.8) | 5.6 (5.6-5.6) | 5.4 (2.8) | 5.7 (5.7-5.8) | +0.1 (2.6) | +0.1 |
| Calcium (mg/d)† | 829.4 (398.4) | 859.0 (852.2-865.7) | 806.7 (375.0) | 836.5 (830.1-842.9) | -13.9 (364.1) | -22.4 |
| Iron (mg/d)‡ | 10.8 (5.8) | 11.8 (11.6-11.9) | 10.2 (5.5) | 11.0 (10.9-11.1) | -0.5 (5.5) | -0.7 |
| Potassium (mg/d)‡ | 2528.8 (1059.1) | 2646.4 (2627.3-2665.5) | 2459.3 (1001.5) | 2552.3 (2534.3-2570.4) | -58.8 (970.5) | -94.1 |
| Magnesium (mg/d)* | 250.9 (115.5) | 266.8 (264.6-268.9) | 248.4 (112.9) | 261.1 (259.0-263.2) | -2.6 (106.7) | -5.7 |
| Sodium (mg/d)‡ | 1998.1 (954.0) | 2139.8 (2120.9-2158.6) | 1764.2 (871.4) | 1890.4 (1872.4-1908.5) | -214.1 (852.7) | -249.4 |
| Saturated Fat (g/d) | 22.8 (14.2) | 25.1 (24.8-25.4) | 22.7 (13.1) | 24.6 (24.3-24.8) | -0.1 (12.5) | -0.6 |
| Total sugar (g/d)‡ | 77.2 (40.1) | 80.9 (80.2-81.6) | 71.3 (36.5) | 75.0 (74.4-75.6) | -4.7 (35.8) | -5.9 |
| % Energy Contribution from Protein‡ | 20.4 (4.1) | 20.6 (20.6-20.7) | 20.7 (4.2) | 21.0 (20.9-21.1) | +0.4 (4.2) | +0.4 |
| % Energy Contribution from Carbohydrates‡ | 44.4 (8.0) | 44.4 (44.4-44.6) | 42.2 (7.5) | 42.1 (41.9-42.2) | -2.1 (8.7) | -2.4 |
| % Energy Contribution from Total Fat‡ | 35.8 (8.1) | 35.1 (35.0-35.3) | 37.3 (6.8) | 37.1 (37.0-37.3) | +1.6 (8.1) | +2.0 |

| | | | | | | |
|---|---|---|---|---|---|---|
| Monounsaturated Fat g/d* | 20.6 (11.6) | 22.3 (22.1-22.5) | 20.8 (11.0) | 22.3 (22.1-22.6) | +0.2 (10.8) | 0.0 |
| Polyunsaturated Fat g/d ‡ | 9.1 (6.6) | 9.9 (9.8-10.1) | 8.5 (5.6) | 9.3 (9.2-9.4) | -0.5 (5.8) | -0.7 |

‡ P-value for change over time was <0.0001; † P-value for change over time was <0.001; *P-value for change over time was <0.05.
